# Ankle Osteoarthritis Aetiology

**DOI:** 10.3390/jcm10194489

**Published:** 2021-09-29

**Authors:** Mario Herrera-Pérez, David González-Martín, Mercedes Vallejo-Márquez, Alexandre L. Godoy-Santos, Victor Valderrabano, Sergio Tejero

**Affiliations:** 1 Foot and Ankle Unit, Orthopedic Surgery and Traumatology Service, Hospital Universitario de Canarias, Carretera de la Cuesta s/n, 38320 Santa Cruz de Tenerife, Spain; davidglezmartin@gmail.com; 2 School of Medicine (Health Sciences), Universidad de La Laguna, Campus de Ofra, s/n, 38071 San Cristóbal de La Laguna, Spain; 3 Musculoskeletal Radiology Unit, Hospital Universitario Virgen del Rocío, Av. Manuel Siurot, s/n, 41013 Sevilla, Spain; mervallejo@gmail.com; 4 Hospital Israelita Albert Einstein, Av. Albert Einstein, 627, Sao Paulo 05652-900, SP, Brazil; alexandrelemegodoy@gmail.com; 5 Orthopaedic Department, Swiss Ortho Center, Schmerzklinik Basel, Hirschgässlein 15, 4051 Basel, Switzerland; vvalderrabano@swissmedical.net; 6 Foot and Ankle Unit, Orthopedic Surgery and Traumatology Service, Hospital Universitario Virgen del Rocío, Av. Manuel Siurot, s/n, 41013 Sevilla, Spain; stejerogarcia@gmail.com; 7 School of Medicine, Universidad de Sevilla, Av. Sánchez Pizjuán, s/n, 41009 Sevilla, Spain

**Keywords:** ankle osteoarthritis, etiology, ankle cartilage, ankle fractures

## Abstract

Ankle osteoarthritis affects 1% of the population and, unlike gonarthrosis or coxarthrosis, is secondary to previous trauma in more than 75% of cases. Another peculiarity of this disease is that it affects a younger and active population, with socio-occupational implications. Mechanical factors, such as incongruity, instability, malalignment, and impacts, which increase stress on isolated areas of the ankle cartilage, have been clearly associated with the development of osteoarthritis. However, we cannot ignore the importance of pro-inflammatory mediators present from the moment of fracture as triggers of the cascade that eventually causes chondrocyte cell death, ultimately responsible for ankle osteoarthritis.

## 1. Introduction

Ankle osteoarthritis is a chronic disease affecting approximately 1% of the population, with an estimated prevalence of 30 cases per 100,000 inhabitants, and corresponds to between 2% and 4% of all patients with osteoarthritis (OA) [1,2]. Cadaver, radiology, and clinical studies indicate that ankle osteoarthritis is less common than knee and hip OA. This is reflected in clinical practice as symptomatic knee osteoarthritis is 8 to 9 times more prevalent than ankle osteoarthritis, and approximately 24 times more total knee work is performed than arthrodesis and arthroplasty combined [3,4]. While its clinical impact on patients has not been considered particularly relevant, ankle OA is very debilitating in advanced stages and can have similar repercussions on quality of life as severe hip osteoarthritis, advanced kidney failure, or congestive heart failure [4,5].

**Figure 1:**
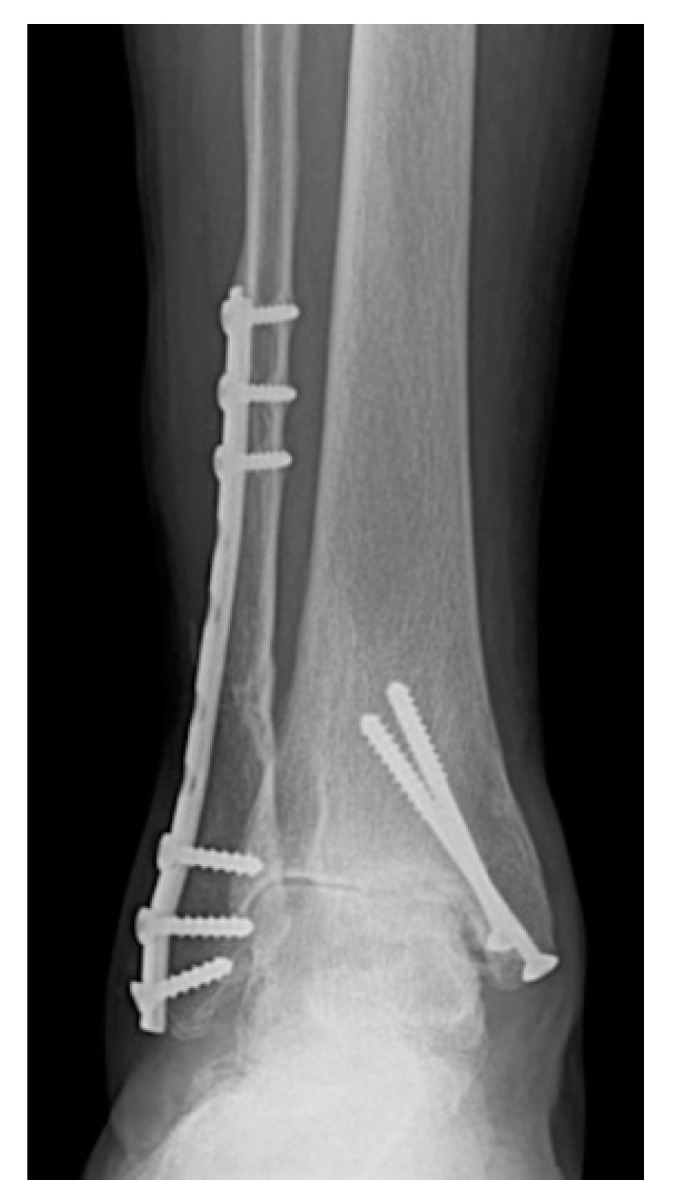
Advanced symmetrical ankle OA secondary to ankle fracture dislocation surgically treated 8 years previously.

There is scant scientific literature available regarding the aetiology of ankle OA; however, there is an apparent consensus that, unlike hip and knee OA, primary ankle osteoarthritis is not the most common aetiology [1,6,7]; only 7% to 9% of cases are classed as idiopathic osteoarthritis and 13% are secondary to other causes (rheumatoid arthritis, haemochromatosis, haemophilia, and osteonecrosis). Therefore, the main aetiology, representing 75% to 80% of all cases, is a traumatic event, with fractures around the ankle (malleolus, distal tibia, talus, and so on) (Figure 1) responsible for 62%, and a further 16% are the result of unresolved chronic ligament injuries, particularly those affecting the lateral collateral ligament (which some authors call “ligamentary ankle osteoarthritis”) [7,8]. Given its predominantly post-traumatic aetiology, patients with ankle OA tend to be younger (18–44 years) than those with other degenerative joint diseases in the lower limbs. They also suffer a more rapid loss of function, with progression to advanced stages of ankle OA occurring 10 to 20 years of onset, although clinical studies show that the initial degenerative changes secondary to an ankle fracture develop within 12 to 18 months of the traumatic injury [7,9].

The present work sets out to describe the main causes of the onset of ankle osteoarthritis.

## 2. Pathophysiology: Peculiarities of Tibiotalar Cartilage

There is a lack of literature covering experimental work around ankle OA; however, some studies have furthered our knowledge of the pathogenesis of joint deterioration [9,10]. It is noteworthy that, despite being a frequently injured joint (fractures, sprains, and so on), clinically relevant ankle OA is less common than in other weight-bearing joints, which can undoubtedly be explained by the anatomical, biochemical, and biomolecular peculiarities of ankle cartilage [9]. Ankle cartilage receives the greatest force per unit area of all hyaline cartilage in the human body (500 N/350 mm^2^ compared with the same force per 1100 mm^2^ or 1120 mm^2^ in the hip or knee, respectively). Thus, ankle cartilage can be subject to three times greater force, yet, as mentioned previously, radiological, and clinical osteoarthritis is less common than in the knee or hip. Furthermore, the load distribution in the ankle differs from in other joints, such as the knee, which means the compressive forces are distributed over a greater area. Ankle cartilage (1–1.62 mm) is thus thinner than knee cartilage (1.69–2.55 mm) [10,11,12]. Biologically, ankle cartilage is believed to have a greater capacity for self-repair than knee cartilage [13]. It is stiffer and less permeable because it produces more water and proteoglycan [9]; in addition, the extracellular matrix improves its load-bearing capacity and reduces its susceptibility to mechanical damage. What is more, chondrocytes in the ankle are metabolically more active than in the knee and exhibit a greater response to anabolic factors influencing cartilage synthesis such as levels of osteogenic protein-1 and C-propeptide of type II collagen. They are also less sensitive to catabolic mediators such as fibronectin and interleukin-1 beta, which inhibit collagen synthesis [10,11,12,13]. Finally, the concentration of matrix metalloproteinases (MMPs), the enzymes responsible for protein degradation in the extracellular matrix, is known to vary between different joints; for instance, MMP-8 levels are significant in knee cartilage, but undetectable in the ankle [13]. Given all these reasons, ankle cartilage is less prone to joint degeneration than that of the knee or hip, but it is highly susceptible to lesions when there is an asymmetric distribution of stresses and forces, as in the case of joint fractures, injuries caused by impacts, or misalignment of the weight-bearing axis [9,14]. Such factors may explain the high correlation between ankle OA and a history of a traumatic event.

## 3. Predisposing Factors for Ankle Osteoarthritis

As mentioned above, most cases of ankle osteoarthritis are secondary to fractures or chronic, unresolved instabilities. Here, we discuss the various factors involved in the development of ankle OA.

### 3.1. Microscopic Cartilage Damage

Circumstances that overload ankle cartilage, either a one-off event (acute impact) or repetitive stress (cumulative contact stress), are known to be a potential cause of clinical osteoarthritis. In 2013, Buckwalter et al. stated that a chronic increase in the mechanical stress on cartilage also causes post-traumatic OA [15]. The proposed mechanism involves an increase in oxidative stress following the release of mitochondria from chondrocytes that are subject to greater contact stress from reactive oxygen species (ROS); mediators of cell damage (chondrocyte death) and extracellular matrix degeneration. Fibronectin fragments released from joint cartilage subjected to excessive loads also promote matrix degradation; inhibition of molecular pathways initiated by these fragments prevents this effect. Additionally, injured chondrocytes release alarmins that activate chondroprogenitor cells, which in turn propagate and migrate to areas of damaged cartilage [13,15]. These cells also release chemokines and cytokines that may contribute to inflammation, which causes progressive cartilage loss. In 2011, Tochigi et al. published an interesting article based on the analysis of cell death in seven ankle specimens collected from amputated patients [16]. They reported that chondrocyte apoptosis occurs mainly in the region of the fracture line, but even more noteworthy was how apoptosis spread spontaneously from the fracture line to apparently healthy areas in the 48 h after the trauma, suggesting that intra-articular mediators of cell damage are released from the injured chondrocytes, and they also affect the healthy chondrocytes.

### 3.2. Macroscopic Cartilage Damage

The direct relationship between osteochondral ankle lesions and future development of ankle OA is controversial; nevertheless, anterolateral talar, posteromedial tibial, and medial malleolar osteochondral injuries have the worst prognosis [5]. Several authors have reported the presence of occult or undiagnosed osteochondral lesions following ankle fractures [17,18], lesions that may explain why a long-term study found that up to 50% of patients with a fractured ankle had suboptimal functional outcomes in terms of persistent pain and a decline in activities of daily living [19]. A recent study observed a high rate of talar osteochondral lesions, both during the acute phase and after 12 months’ evolution (45% and 47%, respectively), among patients who had suffered an ankle fracture, particularly those caused by rotational mechanisms [20]. Consequently, the authors emphasized the need to document the lesions in the acute phase, where arthroscopy would play an essential role in diagnosis and treatment, as indicated in several other studies [17,18,20,21].

### 3.3. Joint Incongruence

It is evident that the presence of an intra-articular step-off favors the development of post-traumatic osteoarthritis in any joint, hence the importance of achieving anatomical reduction of the fracture [17]. This has been demonstrated by McKinley et al. [22], who created an intra-articular step-off in the distal tibia of 10 ankle specimens and observed a substantial increase of up to 300% in the peak contact stress, which could be decisive in the onset of post-traumatic osteoarthritis. Incongruence is closely related to instability; in fact, numerous studies have shown how instability in association with incongruence promotes cell damage to ankle cartilage. McKinley et al. also suggested that instability coupled with incongruence increases the contact stress per surface area by up to 60% in ankle cartilage compared with incongruence alone [23]. In the clinical set, several studies, including a review from 2018 by Verhage et al. [24], argue that postoperative step-off is the most important factor predicting post-traumatic osteoarthritis.

### 3.4. Instability

**Figure 2:**
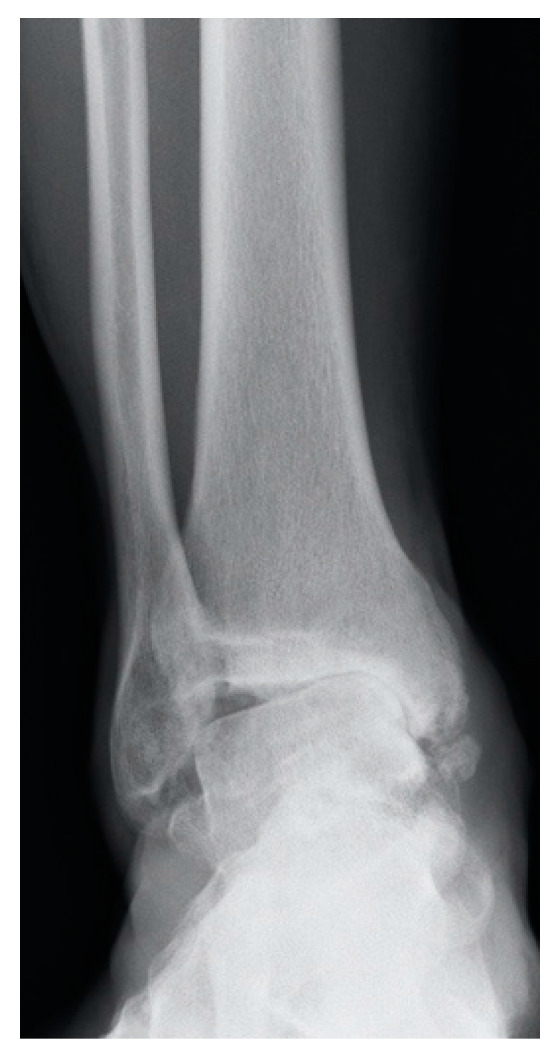
”Ligamentary ankle OA”, advanced asymmetrical ankle OA secondary to chronic unsolved lateral instability.

Instability itself increases the peak joint contact stress, resulting in cartilage deterioration. Authors such as Harrington have shown through radiological and arthroscopic follow-up that ankle instability inextricably leads to osteoarthritis [8]. However, apparent instability is not the only cause of joint damage. Tochigi et al. showed that so-called microinstability following a sprain, intra-articular fracture, or even ligament reconstruction surgery can create isolated peaks of stress in areas of cartilage that are known to lead to joint damage [14]. In clinical practice, many patients with osteoarthritis do not complain of manifest instability and already have advanced OA, so microinstability may play a key causative role in such cases. The term “ligamentary post-traumatic ankle osteoarthritis” (Figure 2) was coined by Valderrabano et al. [25] during an epidemiological study of the main characteristics of this type of ankle OA. It affected approximately 13% of their series, with less clinical impact and a longer mean latency time (34.3 years) than that observed for post-traumatic OA following a fracture. The authors showed, as did Hashimoto and Inokuchi [26], that most instabilities affect the lateral ligament complex, thus increasing stress on the medial joint surface owing to increased pronation and external rotation and producing varus ankle OA.

### 3.5. Malalignment

Tibiotalar osteoarthritis is asymmetrical in almost 70% of cases [27]. In most patients, the deformity underlying the asymmetry is located close to the ankle joint, whether in the supramalleolar region or in the inferior peritalar (subtalar or calcaneal) complex—or both. A history of ankle fracture is very common, which ends up causing uneven load distribution owing to mal union, which, as previously mentioned, is very poorly tolerated by the talar cartilage [28]. Although the origin of most cases of asymmetric ankle arthropathy is close (supra or inframaleolar), it should not be forgotten that it must be explored at the proximal level because deformities at the level of the proximal tibia, knee, or distal femur could be the causes of an asymmetric load distribution on the ankle, which, in turn, results in asymmetric ankle osteoarthritis. In such cases, the problem originally manifesting in the ankle must be resolved surgically away from the ankle [29,30].

### 3.6. Proinflammatory Mediators in Synovial Fluid

Adams et al. reported an interesting finding about the presence of an ambient proinflammatory mediator and promoter of extracellular matrix degradation in the synovial fluid obtained perioperatively from patients undergoing osteosynthesis for an ankle fracture [13]. Interleukins IL-6 and IL-8 and matrix metalloproteinases MMP-2, 3, 9, and 10 were notable and may contribute to the development of post-traumatic OA. The same authors also reported how these proinflammatory factors were still elevated, compared with the healthy contralateral ankle, at 6 months post-surgery, highlighting that joint lavage at the time of fracture may act as a prophylactic [31]. In addition, Furman et al. [32] assure in their work that ankle joint fracture increases local inflammation, as indicated by increased synovitis, increased macrophage infiltration into synovial tissue, and increased synovial fluid concentrations of biomarkers on inflammation. These findings have opened a huge field of research on therapies based on inactivation of proinflammatory substances and cytokines in the treatment of osteoarthritis.

### 3.7. Ageing

Old age is a well-known risk factor for developing osteoarthritis [33,34]. On the other hand, molecular biology studies have shown that the presence of senescent cells in arthritic cartilage supports the idea that the cytokines produced by these cells play a part in the cartilage degeneration cascade that leads to osteoarthritis [35]. The waning capacity of joint cartilage to repair itself as we age is also a relevant factor [36]. Given these reasons, and as pointed out by Valderrabano et al. [2] and especially in the group with primary OA, patients are relatively older (58 years) at the time of diagnosis than those diagnosed with post-traumatic osteoarthritis.

## 4. Conclusions

Osteoarthritis of the ankle is secondary to a periarticular fracture in 75% of cases and chronic, unresolved ligament instability in around 13%. Mechanical factors, such as incongruence, instability, malalignment, and impacts, that increase the stress on isolated areas of ankle cartilage, have been clearly associated with the onset of osteoarthritis. However, we cannot ignore the importance of the proinflammatory mediators present from the moment of fracture as triggers of the cascade that eventually causes the cell damage responsible for osteoarthritis.

## Data Availability

Not applicable.
